# Rare Case of Duodenal Metastasis From Colon Cancer: Review of Literature and Insights on Novel Therapies

**DOI:** 10.1155/crgm/8864636

**Published:** 2025-07-22

**Authors:** Ammar Ismail, William K. B. Boateng, Amira Alnakeb, Youssef Botros, Allan L. Cruz

**Affiliations:** ^1^Jersey City Medical Center, Rutgers New Jersey Medical School, Jersey City, New Jersey, USA; ^2^Faculty of Medicine, Mansoura University, Mansoura, Egypt

## Abstract

Metastasis to the duodenum from colorectal cancer is exceptionally rare and presents significant diagnostic and therapeutic challenges. We describe a 68-year-old female with right colon adenocarcinoma, who developed a duodenal metastasis despite treatment with FOLFOX chemotherapy. Transition to FOLFIRI yielded limited success, emphasizing the need for innovative approaches. Genetic analysis revealed a KRAS G12D mutation, for which targeted therapies are not yet approved. Immunohistochemistry confirmed the gastrointestinal origin of the duodenal mass. Literature indicates that surgical resection can offer curative potential in select cases, although it was not viable here. Emerging KRAS-targeted agents, such as MRTX1133, represent promising options for addressing this mutation. This case underscores the challenges of managing rare metastatic patterns, the potential of personalized therapies, and the necessity for further research into innovative treatments for advanced colorectal cancer. It highlights the importance of developing targeted strategies to improve outcomes for patients with such complex metastatic diseases.

## 1. Introduction

Metastatic colorectal cancer (mCRC) frequently spreads to regional lymph nodes, liver, and lungs. However, metastasis to rare sites such as the duodenum presents a diagnostic and therapeutic challenge, due to its rarity and subtle clinical manifestation. Only a few documented cases reported duodenal involvement in mCRC, which alters both management and prognosis. In this case, we explore the unique progression of colorectal adenocarcinoma with duodenal metastasis and discuss potential therapeutic strategies especially newer agents that work on specific common cancer mutations like KRAS.

## 2. Case Report

**Figure 1:**
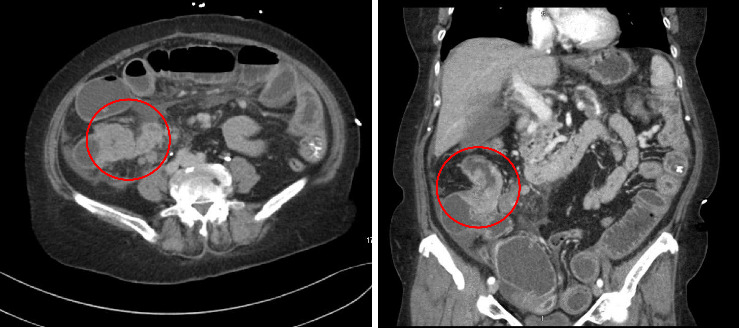
CT abdomen/pelvis with contrast showing ascending colon obstructing mass.

A 68-year-old female with a medical history significant for primary hypertension and type 2 diabetes presented on November 7, 2021, with several days of abdominal pain, distension, constipation, nausea, and vomiting, raising suspicion of bowel obstruction. She reported intermittent abdominal pain, diarrhea, and an unintended weight loss of approximately 30 pounds over the preceding months. Initial imaging via computed tomography (CT) scan of the abdomen and pelvis with contrast revealed an obstructing mass in the ascending colon (Figure 1), mesenteric lymphadenopathy, superior mesenteric vein thrombosis, and moderate ascites.

The patient underwent an emergency right hemicolectomy during the same admission. Histopathological analysis confirmed a well-to-moderately differentiated Grade II adenocarcinoma of the colon classified as pT4a N2b, with 13 out of 16 lymph nodes positive for malignancy. The tumor measured 5.5 cm and showed intermediate tumor budding with necrotic areas. Surgical margins were free of invasive carcinoma. Cytogenetic analysis (Table 1) of the resected mass showed mutations in KRAS_G12D_, NRAS wild-type, BRCA_2_, SMAD_4_, APC, and p53.

**Figure 2:**
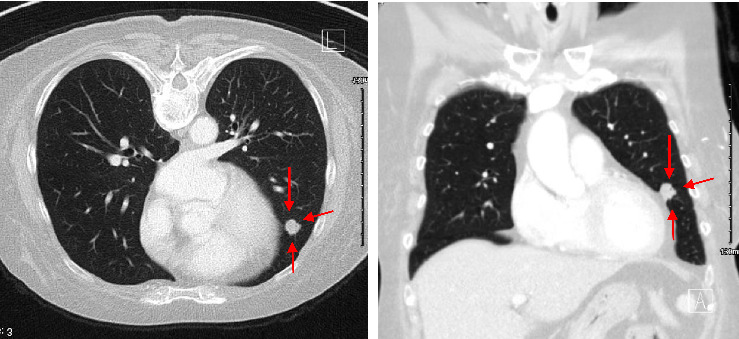
11 mm pulmonary nodule in the left lingula.

A follow-up CT scan of the chest on December 13, 2021, identified an 11 mm lung nodule in the left lingula (Figure 2), and CT guided biopsy on January 6, 2022, confirmed metastatic disease with immunohistochemistry (Table 1): CK7+, CK20−, CDX2+, ER−, PR−, PAX8−, TTF-1−, Napsin A-favoring Gastrointestinal (GI) origin with TNM staging of T4a N2b M1a and stage group IVa according to AJCC 8^th^ edition staging system.

**Figure 3:**
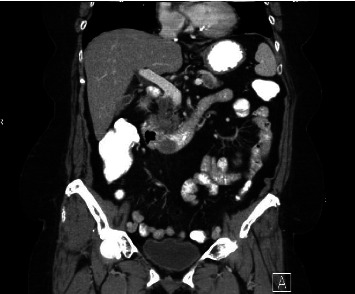
CT abdomen showing 2.5 cm duodenal wall mass.

**Figure 4:**
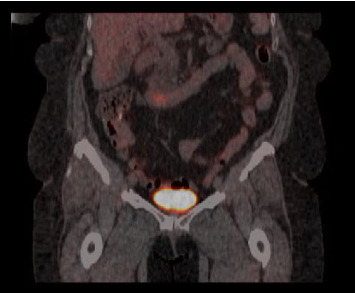
PET scan showing focal fluorodeoxyglucose (FDG) uptake in the third part of the duodenum.

**Figure 5:**
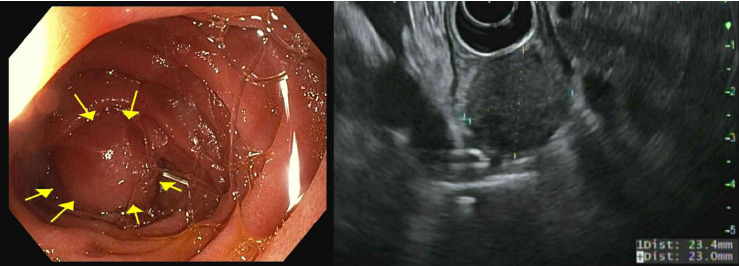
Endoscopic and ultrasound views of the submucosal duodenal mass.

The patient completed six cycles of Folinic acid, Fluorouracil, Oxaliplatin (FOLFOX) chemotherapy from January to March 2022, which initially achieved stable disease on imaging with negative CT chest abdomen and pelvis with contrast in December 2022, March 2023, and June 2023. However, a follow-up CT scan in September 2023 revealed a 2.5 cm duodenal mass (Figure 3), and Positron Emission Tomography (PET) scan showed increased focal fluorodeoxyglucose (FDG) uptake in the same area of the duodenum (Figure 4). An endoscopic ultrasound (EUS) on November 6, 2023, demonstrated a 23 mm submucosal mass in the third part of the duodenum (Figure 5). Fine needle aspiration (FNA) biopsy was performed, and immunohistochemical staining revealed markers consistent with adenocarcinoma of colonic origin, showing positivity for AE1/AE3 and CK7. In contrast, the biopsy was negative for markers typically associated with mesenchymal or neuroendocrine tumors, including CD117, CD34, chromogranin, synaptophysin, and CD56. Tumor TNM staging has changed from M1a to M1b and stage group IVa to IVb.

On January 3, 2024, the patient was initiated on Folinic acid, Fluorouracil, Irinotecan (FOLFIRI), which was planned for 8 cycles. By February 23, 2024, imaging demonstrated minimal reduction in the size of the duodenal mass, and a PET scan on May 13, 2024, showed persistent FDG avidity. Given this finding, treatment was extended to 12 cycles. A repeat EUS on June 26, 2024, showed residual disease, with the duodenal mass reduced to 15 mm, and a repeat FNA biopsy continued to show adenocarcinoma. The patient remains hesitant about surgical intervention, and management discussions are ongoing.

## 3. Discussion

Duodenal neoplasms are rare, whether primary or metastatic. The incidence of primary duodenal cancer is approximately 0.5% of all GI malignancies [1, 2]. In a review of over 11,000 GI tumors, Martin found that 10.8% were derived from the esophagus, 16.4% were derived from the stomach, and 70.3% were derived from the colorectum, but only 2.4% were derived from the small bowel [3]. The largest previous series of small bowel adenocarcinoma between 1973 and 1991 found an incidence of 5.7 cases per million population per year in whites and 7.5 cases per million in blacks over that period [4]. Metastasis to the duodenum is also rare. In a retrospective study collecting data about metastatic cancers to the GI tract, the duodenum was a rare destination occurring in approximately 5% while the stomach and colorectum were the most common sites of metastasis in the GI tract accounting for almost 40% each [5]. In our analysis, the CK7 positivity and CK20 negativity observed in both the lung and duodenal masses would typically suggest an upper GI origin. However, considering the patient's history of a resected pT4a colon adenocarcinoma, we believe colorectal metastasis remains the most likely diagnosis despite the unusual CK7+/CK20− profile [6]. The American Society of Colon and Rectal Surgeons suggests PET/CT as an option for patients with known metastatic colon cancer who are being considered for curative resection as the identification of otherwise unrecognized metastatic disease may alter the treatment plan. In our case, PET/CT was not initially performed until duodenal mass was detected on contrast enhanced CT abdomen [7].

Few publications have documented similar cases of duodenal metastasis from colorectal cancer. The earliest was reported in 2013 by Brahmbhatt et al. in a patient who presented with recurrent adenocarcinoma of the colon presenting as a duodenal mass with partial gastric outlet obstruction which was managed by surgical resection, considering that surgery followed by adjuvant chemotherapy is the only curative option [8]. In 2017, Stamopoulos et al. also reported a case of duodenal metastasis that was surgically managed, raising the question of whether simple microscopic and macroscopic margin-negative resection (R0) is oncologically equivalent to radical resection [9]. In 2023, Chehal et al. presented a similar case who initially responded well to distal gastric duodenectomy and adjuvant chemotherapy (FOLFOX). However, he had a subsequent relapse and developed metastasis in the liver, lung, and multiple lymph nodes, which improved with multiple cycles of FOLFIRI. In this case, genomic analysis was reported as KRAS, NRAS wild, and BRAF wild [10].

First-line backbone chemotherapy regimens used in mCRC were divided into three groups: mono (5-Fluorouracil/Leucovorin, Capecitabine, Tegafur-uraci/Leucovorin, or S-1 monotherapy), doublet (e.g., FOLFOX or FOLFIRI), and triplet (FOLFOXIRI) [11].

Tumor cytogenetic analysis can aid in predicting prognosis, and guide choice of therapeutic agents. In colorectal cancer patients, mutations in KRAS, p53, or SMAD4 were associated with a higher risk of distant metastasis [12]. KRAS mutations are one of the most dominant mutations in colorectal cancer and are associated with poor prognosis and drug resistance [13]. Although KRAS mutations were discovered in the 1980s, drugs that target these mutations have not developed and direct drug targeting remains challenging due to the lack of classic drug binding sites [11].

Studies have reported that around 40% of mCRC have KRAS mutations. Among these, mutations in KRAS_G12D_ were most common (36%), followed by KRAS_G12V_ (21.8%), KRAS_G13D_ (18.8%), and KRAS_G12C_ in around 17% [14–16]. Although a less common mutation than KRAS_G12D_, the identification and subsequent clinical success of irreversible KRAS_G12C_ inhibitors that occupy the induced switch II pocket has been a very important breakthrough [17]. KRAS_G12C_ inhibitors (e.g., Sotorasib or Adagrasib) in combination with EGFR inhibitors (e.g., Cetuximab or Panitumumab) are studied and being used in the treatment of mCRC with KRAS G12C mutations [18, 19]. There are currently no FDA-approved selective KRAS_G12D_ inhibitors; however, MRTX1133 is currently being studied as a noncovalent, potent, and selective inhibitor of KRAS_G12D_. MRTX1133 was shown to suppress KRAS_G12D_ signaling in cells and in vivo, and its antitumor benefit was demonstrated in a murine animal model and further research can be promising [17].

## 4. Conclusion

This rare case of duodenal metastasis from colorectal adenocarcinoma emphasizes the complexity of mCRC, particularly in such an uncommon site. FOLFOX and FOLFIRI regimens are typically used in managing stage IV colorectal carcinoma. Despite initial response and disease control with the FOLFOX chemotherapy regimen, the observed metastatic duodenal mass, and minimal response to the subsequent transition to FOLFIRI therapy, highlights the need for further insights into the management of such complex cases. Literature about such metastasis, although limited, suggests that surgical resection of isolated duodenal metastases can be effective but remains an option only for select patients who are surgical candidates and willing to undergo surgery. KRAS_G12C_ inhibitors are being used and are effective for KRAS_G12C_. However, KRAS_G12D_ mutations, which are more common, still have no approved drugs, and new studies are now experimenting with selective KRAS_G12D_ inhibitors. This case underscores the need for further research into innovative treatments for rare metastatic patterns.

## Figures and Tables

**Table 1:** Pathological findings in 3 different tumor sites.

	Primary tumor	Lung nodule	Duodenal nodule
Date of excision	11/07/2021	01/06/2022	11/06/2023

Histological type	Adenocarcinoma	Adenocarcinoma	Adenocarcinoma

Immunohistochemistry	MLH1 (+ve) MSH2 (+ve) MSH6 (+ve) PMS2 (+ve)	CK7 (+) CK20 (−) CDX2 (+) ER (−) PR (−) PAX8 (−) TTF-1 (−) Napsin A (−)	AE1/AE3 (+) CK7 (+) CD117 (−) CD34 (−) Chromogranin (−) Synaptophysin (−) CD56 (−) Ki67∼20%

Cytogenetic analysis (mutations)	KRAS_G12D_ NRAS wild-type BRCA_2_ SMAD_4_ APC p53	—	—

## Data Availability

The data that support the findings of this study are available from the corresponding author upon reasonable request.
